# Sudden shortening of the paced AV delay: is this normal pacemaker function?

**DOI:** 10.1007/s12471-014-0644-7

**Published:** 2015-01-14

**Authors:** Arnold Pinter, Paul Dorian

**Affiliations:** St. Michael’s Hospital, University of Toronto, 30 Bond Street, M5B 1W8 Toronto, ON Canada

A 74-year-old female patient underwent a Medtronic KDR601 dual chamber pacemaker implantation for syncope due to sinus node disease. The ventricular and atrial passive fixation pacemaker leads were positioned in the right ventricular apex and right atrial appendage, respectively. The sensing and capture threshold values were satisfactory in both chambers. The device was programmed to DDDR mode 60–130 ppm with both the upper tracking rate and maximum sensor rate at 130 ppm, paced AV delay at 200 ms, and sensed AV delay at 180 ms. Postoperative chest X-ray showed normal pacemaker lead positions and no sign of complications.

The postoperative 12-lead ECG showed mostly AV sequential pacing, with occasional spontaneous P waves followed by native QRS (Fig. 1). The third complex of the tracing shows a shortened paced AV delay of 110 ms. Is this normal pacemaker function?

**Fig. 1:**
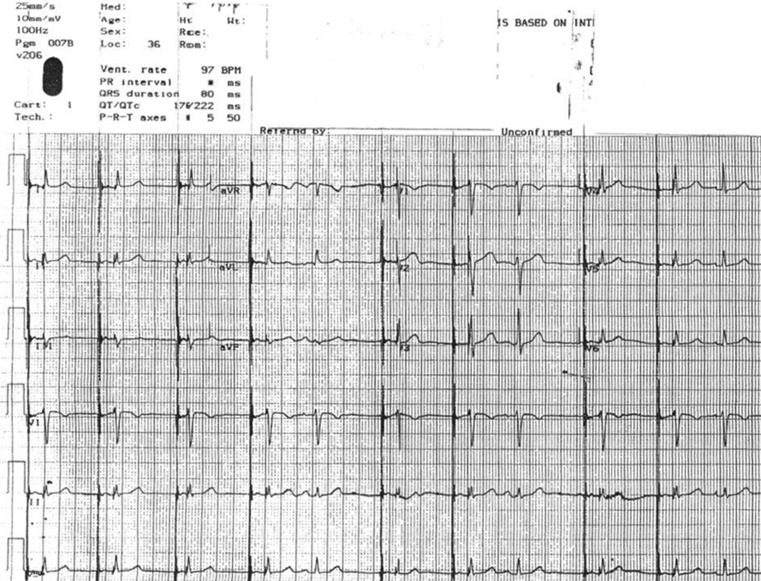
Postoperative standard 12-lead ECG (1 mV/10 mm, 25 mm/s paper speed)

**Answer**

You will find the answer elsewhere in this issue.

## Conflict of interest

None of the authors have any conflict of interest related to this report.

